# Metabolite Profiling and Classification of Highbush Blueberry Leaves under Different Shade Treatments

**DOI:** 10.3390/metabo12010079

**Published:** 2022-01-14

**Authors:** Yaqiong Wu, Hao Yang, Zhengjin Huang, Chunhong Zhang, Lianfei Lyu, Weilin Li, Wenlong Wu

**Affiliations:** 1 Jiangsu Key Laboratory for the Research and Utilization of Plant Resources, Institute of Botany, Jiangsu Province and Chinese Academy of Sciences, The Jiangsu Provincial Platform for Conservation and Utilization of Agricultural Germplasm, Qian Hu Hou Cun No. 1, Nanjing 210014, China; ya_qiong@126.com (Y.W.); hzj90@cnbg.net (Z.H.); chzhang0714@163.com (C.Z.); njbglq@163.com (L.L.); 2 Co-Innovation Center for Sustainable Forestry in Southern China, Nanjing Forestry University, 159 Longpan Road, Nanjing 210037, China; yanghao_19940720@163.com

**Keywords:** blueberry, shading, leaf metabolite, multivariate statistical analysis, metabolism pathway

## Abstract

Blueberry belongs to the genus *Vaccinium* L. in the Ericaceae and is an economically important shrub that produces small berries that are rich in nutrients. There were differences in the appearance of blueberry leaves under different shade treatments. To explore the differences in metabolites in blueberry leaves under different shading treatments, nontargeted liquid chromatography–mass spectrometry (LC–MS) metabonomic analysis was performed. Different shade intensities resulted in significant differences in the contents of metabolites. A total of 6879 known metabolites were detected, including 750 significantly differentially expressed metabolites, including mainly lipids and lipid-like molecules and phenylpropanoid and polyketide superclass members. Based on a Kyoto Encyclopedia of Genes and Genomes (KEGG) pathway analysis, the flavone and flavonol biosynthesis pathways were the most significantly enriched. The results of this study provide a reference and scientific basis for the establishment of a high-quality and high-yield shaded blueberry cultivation system.

## 1. Introduction

Light, as an important ecological factor, affects the growth and development of plants. Light often acts on plants in the form of environmental signals, causing plants to undergo different physiological, morphogenetic and metabolic changes to adapt to different light environments [1,2,3]. Therefore, light conditions are often considered to be one of the environmental factors most closely related to plant development and secondary metabolism [4]. Plants of the same kind have different light environment requirements at different growth stages, and people often adopt a variety of technical measures to adjust the light intensity to meet the needs of plant growth [5]. Jeltsch et al. [6] found that the distribution and increase in carbon with plants were significantly affected under the inhibition of strong light. Light intensity plays contrasting roles in regulating metabolite compositions in *Brassica rapa* var. *parachinensis* [3]. The plant growth, biomass accumulation, pigment content and phenolic content of *Cyclocarya paliurus* were significantly affected by different light quality treatments [2]. Shading is a commonly used technique to regulate light intensity and can reduce the photosynthetically active radiation of plants, thus affecting photosynthesis and photomorphogenesis [7,8,9]. Previous studies have pointed out that plants can adapt to changes in the light environment by increasing their specific leaf area, reducing leaf thickness, increasing photosynthetic pigment contents and increasing the absorption of mineral elements [10]. In addition, previous studies have shown a relationship between plant secondary metabolism and light intensity. The carbon/nutrient (C/N) balance hypothesis holds that under the condition of sufficient nutrients, shading can inhibit photosynthesis and decrease C/N in plants, resulting in a decrease in C-based phenols (including flavonoids) and terpenes [11]. Spatiotemporal shading can regulate anthocyanin, proanthocyanidin and sucrose accumulation in black soybean seeds [7]. Due to the important role of plant secondary metabolites in improving plant stress resistance and coordinating the relationship with the environment, their synthesis and accumulation are more easily affected by environmental conditions than those of primary metabolites [12].

Blueberry is an important small berry fruit tree in economic forests [13,14]. Blueberry fruit is rich in nutrients, such as phenols, anthocyanins, mineral elements and vitamins. Moreover, compared with traditional fruits, such as grapes, apples and tomatoes, blueberry fruits contain more antioxidants, among which anthocyanins are the most effective [15,16]. Eating these antioxidants greatly benefits human health and can effectively prevent a variety of diseases. At present, it has been confirmed that blueberries contain antioxidants, antihypertensives, cardiovascular protectants and other substances that prevent chronic diseases and perform other functions [17,18]. Therefore, blueberries are listed as one of the top five health foods by the Food and Agriculture Organization of the United Nations (FAO) and are known as “golden berries” [19]. With blueberry industrialization, the growth and accumulation of secondary metabolites in blueberries are the top two main concerns. While growth is the basis of yield formation, there are few studies on the effects of environmental factors, especially shade treatment, on the growth and secondary metabolites of blueberry.

Metabonomics technology can be used to study the types and contents of metabolites in the same plant at different developmental stages or in different tissues and organs [20,21]. For example, Wu et al. [21] analysed the kernels of *Styrax tonkinensis* at different developmental stages by metabonomics and fully elucidated the metabolic substances and enriched pathways related to oil synthesis during seed development. Bai et al. [22] comprehensively investigated the phytochemical constituents and multiple biological activities of different parts of tree peony fruits harvested from *Paeonia ostii* and *Paeonia rockii.* Guo et al. [23] used metabonomics and transcriptomics to analyse the similarities and differences of phenylpropanoid and flavonoid synthesis-related genes and metabolites in young and mature leaves of *Ginkgo biloba* and provided useful information for the practical application of ginkgo leaves in the pharmaceutical industry. The diversity of metabolites in plants makes them an ideal target for studying the regulatory mechanisms of biosynthesis [24]. With the progress of science and technology, metabonomics has become a widely used tool in systems biology and molecular breeding, especially for high-throughput analysis, chromatography and the analysis of metabolites; the availability of bioinformatics tools and databases has also improved [25,26,27,28].

In this study, different shading treatments were used in the cultivation of potted blueberry seedlings. The goal was to deeply understand the effects of different shading intensities on the metabolites of blueberry leaves to determine which appropriate shading intensity is conducive to the growth of blueberries and the accumulation of metabolites. To achieve this goal, (i) we identified all metabolites in blueberry leaves; (ii) the metabolic substances were classified, and the change trends of the main metabolic substances under different shading intensities were determined; and (iii) the differentially expressed metabolites (DEMs) were identified across different shading treatments, and the main DEMs were enriched by a Kyoto Encyclopedia of Genes and Genomes (KEGG) pathway analysis. Overall, the aim of this study was to determine the optimum conditions for blueberry leaf growth and metabolite accumulation under different light intensities to provide a reference and scientific basis for the establishment of a high-quality and high-yield shaded blueberry cultivation system. In addition, this research provides strong evidence for optimizing the cultivation conditions and intensive management of blueberry in the summer.

## 2. Results

### 2.1. Phenotypic Observation and Leaf Colour Analysis

**Figure 1:**
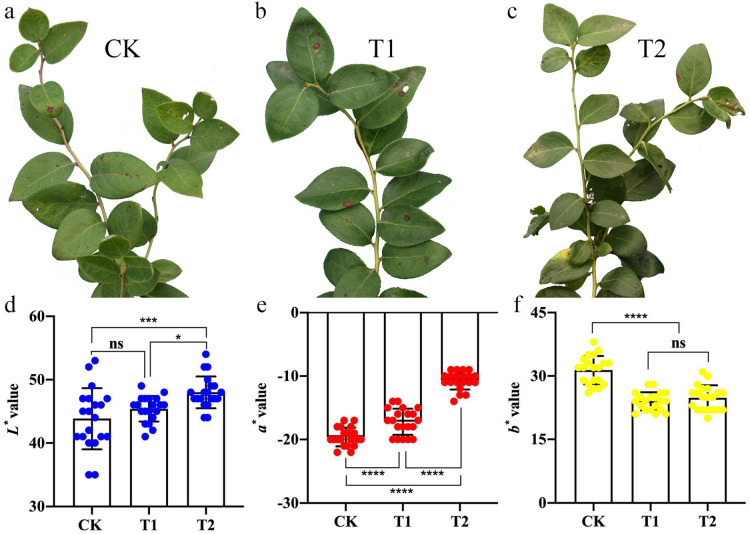
Phenotypic observations (**a**–**c**) and colour parameters (**d**–**f**) of blueberry leaves. *, *** and **** represents a significant correlation at the 0.05, 0.001 and 0.0001 level, respectively. The ns represents no significant correlation.

**Figure 2:**
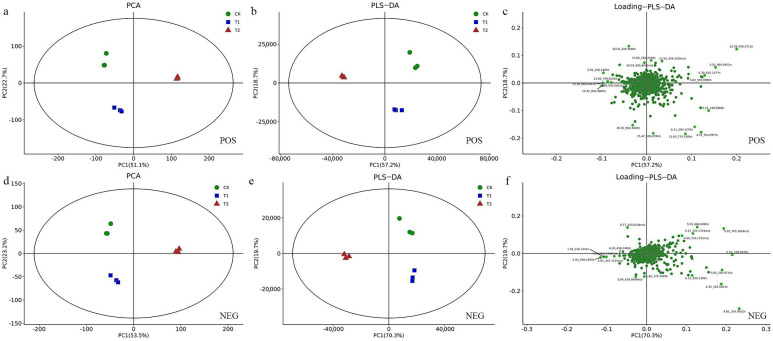
Multivariate statistical analysis of blueberry samples in positive and negative ion modes. Principal component analysis in positive (**a**) and negative ion modes (**d**); partial least-squares discriminant analysis score chart in positive (**b**) and negative ion modes (**e**); and loading plot in positive (**c**) and negative ion modes (**f**).

Shading treatment was carried out using a one-layer black shading net (T1, 50% shading), two-layer black shading net (T2, 80% shading) and no shading (CK, total light). After shading for 2 months, there were some differences in the appearance of blueberry leaves under different treatments (Figure 1). Compared with the leaves of the CK group, the leaves of the T1 group were greener (Figure 1a,b), and the leaves of the T2 group were more yellow–green (Figure 2c). Furthermore, to explore the difference in blueberry leaf colour among the CK, T1 and T2 treatment groups, the values of the colour parameters L* (colour brightness), a* (+red/−green degree) and b* (+yellow/−blue degree) were measured and analysed (Figure 1d–f). The results showed that the L* and b* values of blueberry leaves in the three groups were greater than 0, indicating that their colour was brighter. The L* and b* values of the blueberry leaves were significantly different between the CK and T2 groups. The order of the L* values were as follows: T2 > T1 > CK (Figure 1d); that of the b* values was as follows: CK > T1 > T2 (Figure 1f). The a* values of blueberry leaves in the CK, T1 and T2 groups were less than 0, indicating that their colour was green, and there were very significant differences among the different groups (Figure 1e).

### 2.2. Multivariate Statistical Analysis

The base peak chromatogram (BPC) is a map obtained by continuously depicting the intensity of the strongest ion in the mass spectrum at each time point. Figures S1 and S2 are BPC diagrams of 9 samples in positive and negative ion modes, respectively. According to the qualitative metabolite results, there were 24,529 substance peaks in blueberry leaves from the CK, T1 and T2 groups (15,218 in positive ion mode and 9311 in negative ion mode), while the total number of known metabolites was 6879, including 4727 in positive ion mode and 2152 in negative ion mode. We used unsupervised principal component analysis (PCA) to observe the overall distribution among samples and the stability of the whole analysis process. The elliptical region represents the 95% confidence interval. By observing the dispersion of samples, it was concluded that the stability and reliability of the instrumental analysis were good, and the samples between different groups were relatively far away on the abscissa and ordinate score diagrams. The degree of variation was large, and there may have been significant differences. The variability within three biological repeat samples in the group was small, which showed that biological repeatability in the same group was good (Figure 2a positive ion mode, and 2d negative ion mode). Supervised partial least-squares discriminant analysis (PLS-DA) with the addition of grouping variables can compensate for the deficiencies of the PCA. In positive ion mode, the parameter R^2^X (cum) = 0.90, the interpretation rate R^2^Y (cum) and the prediction rate Q^2^ (cum) were close to 1, indicating that the PLS-DA model can better explain and predict the differences in samples among the different groups (Figure 2b). In negative ion mode, the parameters R^2^X (cum) = 0.95 and Q^2^ (cum) = 0.99 indicated that the model established by this method was stable and reliable (Figure 2e). In addition, loading plots can be used to determine the intensity of the impact of metabolites in different comparisons. A load farther away from 0 indicates that the variable has a greater impact on the component, and a load closer to 0 indicates that the variable has a lesser impact on the component (Figure 2c positive ion mode, and 2f negative ion mode).

### 2.3. DEMs Analysis

**Figure 3:**
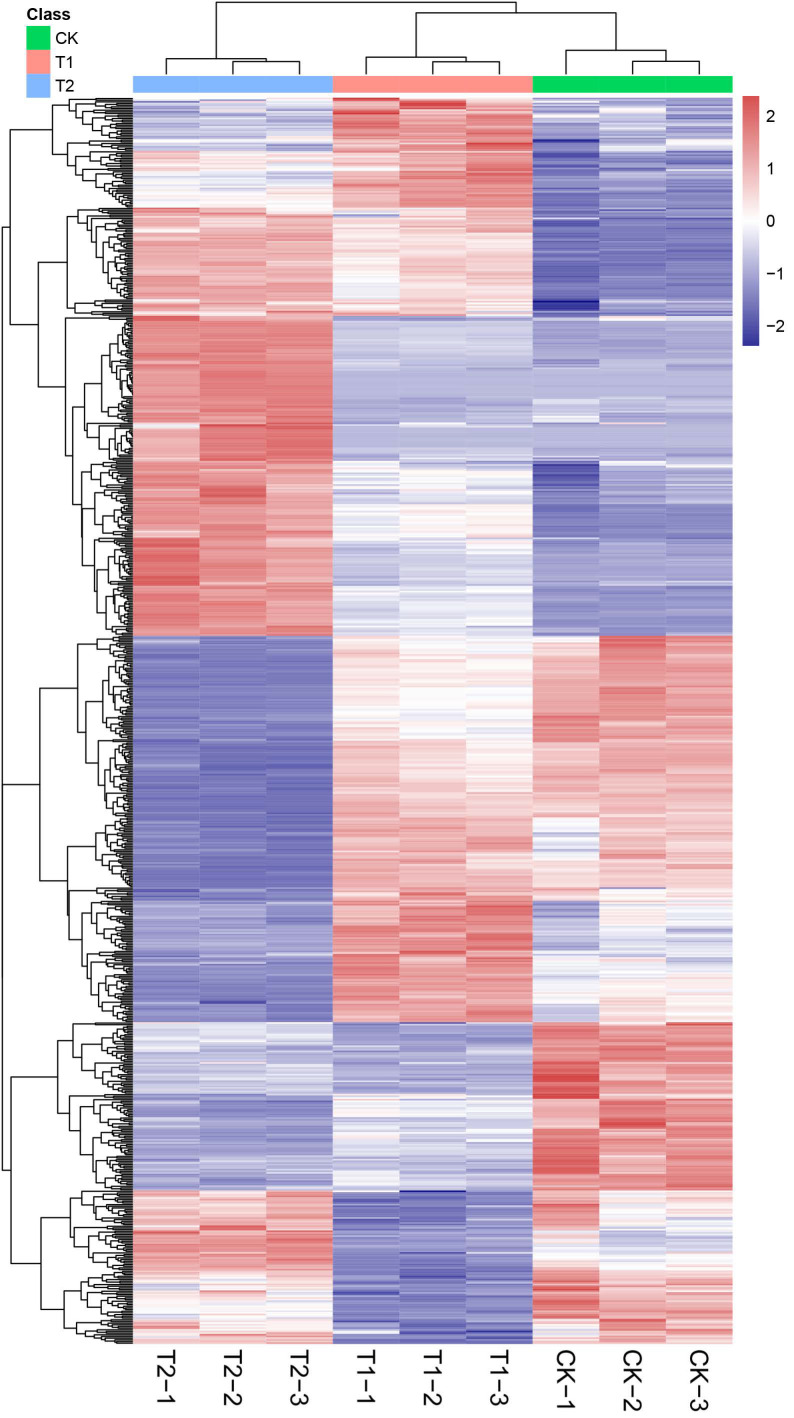
Vertical and horizontal cluster heatmap of significantly differentially expressed metabolites (DEMs) in blueberry leaves. The abscissa represents the sample name, and the ordinate represents the DEMs.

**Figure 4:**
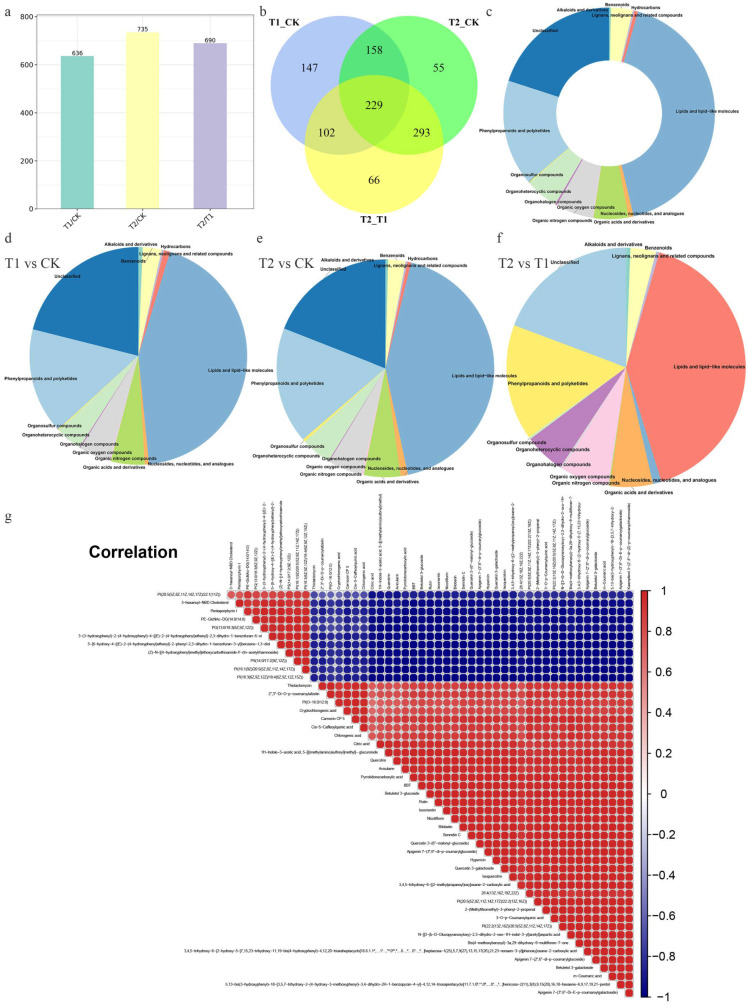
Statistical and analytical charts of differentially expressed metabolites (DEMs). (**a**) DEMs in the different comparisons. (**b**) The number of DEMs in the different comparisons. (**c**) Classification of all DEMs classification. (**d**–**f**) DEMs in different superclasses between the T1 and CK groups, T2 and CK groups, and T2 and T1 groups, respectively. (**g**) Correlation analysis of the top 50 DEMs.

We used the combination of multidimensional analysis and one-dimensional analysis to screen the DEMs between groups and further verified whether the DEMs between groups were significant by T test. To more intuitively show the relationships and the expression differences of metabolites between different samples, we performed hierarchical clustering based on the expression of all significant DEMs (750) (Figure 3; Table S1). The colours from blue to red indicate the expression of metabolites from low to high; that is, the redder colour indicates that the expression of DEMs is higher. In addition, based on the specific numbers of DEMs in each comparison group, the highest number of DEMs was found between the T2 and CK groups, at 735, while there were 636 DEMs between the T1 and CK groups, and 690 DEMs between the T2 and T1 groups (Figure 4a). Interestingly, 229 DEMs were common to all comparison groups (Figure 4b). We classified all DEMs according to their chemical properties and obtained 150 unclassified DEMs, 314 DEMs belonging to lipids and lipid-like molecules, and 122 DEMs belonging to phenylpropanoids and polyketides (Figure 4c). The remaining DEMs were distributed among 11 different superclasses, and the number of DEMs in each superclass was no more than 50. To better understand the classification of DEMs between different groups, we performed statistical analysis on the classification of DEMs in each comparison group. There was only one DEM in the three superclasses of organic nitrogen compounds, organohalogen compounds, and organosulfur compounds in the T1 and CK comparison (Figure 4d). In the T2 and CK comparison, the number of DEMs in the six superclasses of alkaloids and derivatives, hydrocarbons, organic nitrogen compounds, organohalogen compounds, organosulfur compounds, lignans, neolignans and related compounds was less than 5 (Figure 4e). Between the T2 and T1 groups, the most abundant DEMs were lipids and lipid-like molecules (281), followed by unclassified molecules (133) (Figure 4f). Since the greatest difference in the expression abundance of DEMs was found between the T2 and CK groups, the correlation analysis of the top 50 DEMs in this group showed that there was a positive correlation between six common flavonoids (quercitrin, rutin, quercetin 3-(6″-malonyl-glucoside), quercetin-3-galactoside, isoquercitrin, and kaempferol 3-(2″,4″-di-(Z)-p-coumaroylrhamnoside)), and the correlation coefficients were greater than 0.97 (Figure 4g).

### 2.4. KEGG Pathway Enrichment Analysis of DEMs

**Figure 5:**
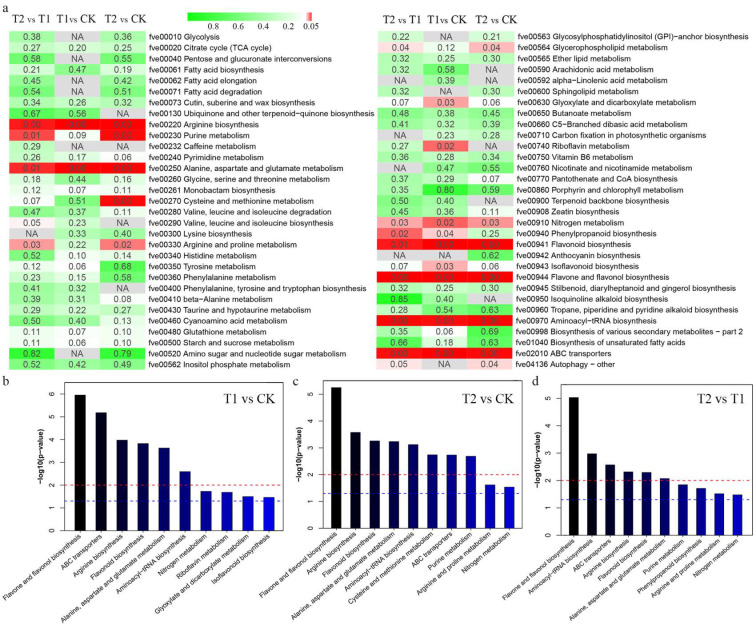
KEGG pathway enrichment analysis based on differentially expressed metabolites (DEMs). (**a**) The main enriched metabolic pathways of the DEMs. (**b**–**d**) The top 10 enriched KEGG pathways. The horizontal coordinate is the name of the pathway, the vertical coordinate is the −log10 (*p* value) and the two horizontal dashed lines represent the positions of *p* < 0.01 and *p* < 0.05.

The significant DEMs were annotated by the KEGG database, and the main enriched metabolic pathways of the DEMs were analysed. The results showed that the DEMs were enriched in 62 KEGG metabolic pathways, of which 41 pathways were found across all comparisons, and only 3 pathways existed in only one comparison (ko00232 caffeine metabolism, ko00592 alpha linolenic acid metabolism and ko00942 anthocyanin biosynthesis) (Figure 5a). We analysed the top 10 pathways with significant KEGG enrichment in the different comparisons and found that the flavone and flavonol biosynthesis pathways were the most significantly enriched (Figure 5b–d). In addition, the enrichment of six pathways, namely, flavonoid biosynthesis, ABC transporters, arginine biosynthesis, aminoacyl-tRNA biosynthesis, nitrogen metabolism, and alanine, aspartate and glutamate metabolism, ranked in the top 10 in each comparison.

### 2.5. Analysis of the Metabolites in Six Pathways

**Figure 6:**
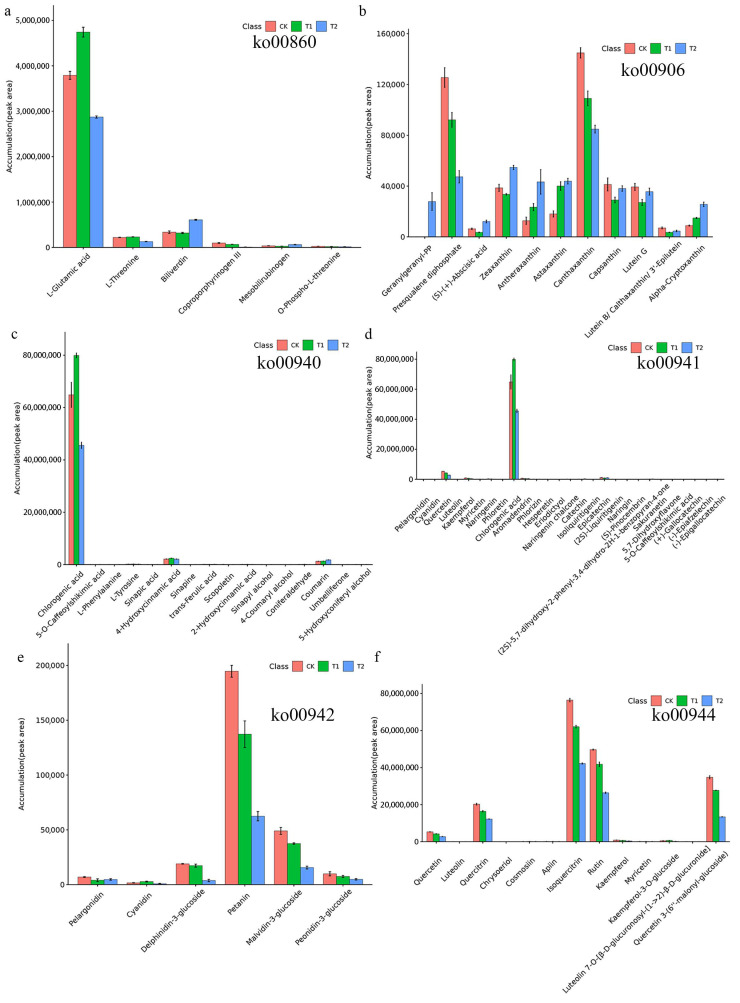
Analysis of the metabolites enriched in six important pathways. (**a**) Porphyrin and chlorophyll metabolism pathway; (**b**) carotenoid biosynthesis pathway; (**c**) phenylpropanoid biosynthesis; (**d**) flavonoid biosynthesis; (**e**) anthocyanin biosynthesis pathway; and (**f**) flavone and flavonol biosynthesis pathway.

There were six metabolites (L-glutamic acid, L-threonine, biliverdin, coproporphyrinogen III, mesobilirubinogen and O-phospho-L-threonine) in the porphyrin and chlorophyll metabolism pathway (ko00860), of which the content of L-glutamic acid was the highest, especially in the T1 group (Figure 6a). In the carotenoid biosynthesis pathway (ko00906), 11 metabolites were annotated. The contents of canthaxanthin and presqualene diphosphate were relatively high, and the contents of (S)-(+)-abscisic acid and lutein B/calthaxanthin/3′-epilutein were relatively low (Figure 6b). The chlorogenic acid content was relatively high in the phenylpropanoid biosynthesis (ko00940) and flavonoid biosynthesis (ko00941) pathways (Figure 6c,d). Moreover, a total of 6 metabolites were annotated into the anthocyanin biosynthesis pathway (ko00942) (Figure 6e). The trends in the contents of petanin, malvidin-3-glucoside, peonidin-3-glucoside and delphinidin-3-glucoside in each group were CK > T1 > T2. In the flavone and flavonol biosynthesis pathway (ko00944), 13 metabolites were found, with isoquercitrin and rutin having the highest content; the trend for the content of these two metabolites in each group was CK > T1 > T2 (Figure 6f).

## 3. Discussion

With the expansion of blueberry planting areas, large-scale breeding of blueberry seedlings is important to the healthy development of the blueberry industry. To cultivate blueberry seedlings with strong growth, high survival rates and good quality, the effects of different shade treatments on blueberry growth and physiological metabolite indexes were studied to determine the shading degree suitable for blueberry growth in the summer (T1: 50% shading) and to provide a technical reference for the scientific cultivation, management and large-scale breeding of blueberry.

As one of the most important environmental factors, light is indispensable for plant compound biosynthesis. Key factors related to light include the intensity (quantity), photoperiod (duration) and light quality (frequency or wavelength) [29]. The results showed that shading had important effects on leaf gas exchange, leaf pigments and secondary metabolites in *Polygonum minus* Huds., an aromatic medicinal herb [30]. Similarly, in a study on the effect of light intensity on the metabolite content of *Centella* plants, it was also found that a 50% shading treatment would reduce the contents of triterpenoids, flavonoids and chlorogenic acid in the leaves [31], and in blueberries [32] and *Berberis microphylla* G. Forst, consistent results were also found for anthocyanin synthesis [33]. However, some studies reported that moderate shading could induce the accumulation of plant secondary metabolites [34] and increase the anthocyanin content [35]. Light plays an important role in plant growth and development, and plants have different stress responses to light [36,37,38]. This study found that compared with the control, the blueberry leaves under the T1 shading treatment were significantly darker and greener than those under other treatments (Figure 1a,b). This result may have been because appropriate shading can increase the chlorophyll contents in the leaves [39], which can cause the leaves to capture more photons and enhance photosynthesis under weak light conditions. However, the colour of blueberry leaves treated with excessive shading was significantly lighter (Figure 1c), which was similar to the results of Chen et al.’s study [40]. It is inferred that moderate shading can increase the chlorophyll content of blueberry leaves, but excessive shading will lead to the decomposition of chlorophyll. Some studies have also shown that shading may balance the source–sink gradient, thereby reducing feedback inhibition, leading to photoinhibition and oxidative stress [41]. Moderate shading alleviates sink limitation by preventing excessive starch accumulation and increasing leaf sucrose levels [41]. The relationship between plant structure and the environment plays an important role in exploring the stress resistance of plants. Most related studies have been performed on leaves. Usually, plant leaves are the most common organ in which to study photosynthesis and changes in the external environment. Under different shading environments, the changes in plant growth and morphological characteristics were the most obvious. These adaptive changes are conducive to improving plant survival, fitness and competitiveness.

Metabolomics is a comprehensive analysis of the whole metabolome under specific experimental conditions. It ultimately defines the chemical phenotype of organisms at a certain time point [42]. This chemical phenotype, the metabolome, is a collection of all metabolites of an organism and is the result of genomic functioning in a specific environment [43]. Therefore, both genomic and environmental changes will eventually appear in the metabolome and finally result in gene and protein expression. Plant metabolomics aims to comprehensively and in an unbiased manner determine the response of metabolite concentrations in organisms to interference by biotic or abiotic factors [44,45]. Wu et al. [21] analysed the metabolites of kernels at different developmental stages of *Styrax tonkinensis* through metabolomics, obtaining 187 and 1556 metabolites through GC–MS and LC–MS, respectively. Zou et al. [46] conducted extensive targeted metabolomics analysis based on LC–MS/MS for loquat, and a total of 536 metabolites were identified. Among them, 193 metabolites (including 7 carbohydrates, 12 organic acids and 8 amino acids) were different between the different cultivars. Through enrichment analysis, it was also found that there were significant differences in the phenolic pathways between different cultivars [46]. In this study, nontargeted LC–MS analysis was used to obtain a total of 6879 known metabolites, including 2152 in negative ion mode and 4727 in positive ion mode. There were significant differences in the number of metabolites between different species/tissues.

The synthesis and accumulation of secondary metabolites in plants are induced by certain environmental conditions [47]. As a result, the secondary metabolite synthesis pathway is distributed in different organs, tissues and cells of plants and is regulated by developmental processes. Sometimes the environmental conditions that result in the highest flavone content may not be the most suitable growth conditions for plants. From the perspective of plant cultivation, selecting a sub-suitable ecological environment with certain environmental pressures is sometimes used to balance the cultivation and growth of some medicinal plants and the accumulation of secondary products [48]. Correlation analysis can help to measure the correlation between significant DEMs and further reveal the relationships between metabolites in the process of biological state change. Herein, we found that there was a positive correlation between six common flavonoids, and the correlation coefficients were greater than 0.97 and *p* value were less than 0.05, indicating that when one metabolite increased, the other metabolites with a very significant correlation also increased. Several studies have found that after shading, the polyphenol content of tea will decrease and the amino acid content will increase to reduce the phenol:ammonia ratio and improve the quality of tea [49]. However, the results of this study are not completely consistent. The changes in the contents of various polyphenols in different groups were not exactly the same, indicating that there may be competition for common substrates among various phenolic metabolites. Light energy beyond the acceptable range of plant light response centres can lead to light stress and photoinhibition [50]. In addition to possibly affecting blueberry growth and fruit flavour, flavonol glycosides are effective attenuators of sunlight, so they are considered to play a key role in plant photoprotection [51,52]. In this study, after shading treatment, the content of most flavonoid (alcohol) glycosides decreased further with an increase in the degree of shading (Figure 6e,f), which is similar to the results of Wang et al.’s [53] study. KEGG pathway enrichment analysis showed that the flavone and flavonol biosynthesis pathways were the most significantly enriched in this study among the different comparisons. It is suggested that changes in this product from this pathway may be important in the response of blueberry leaves to different shading stresses. Several studies have indicated that flavonoids play a certain role in protecting plants from light [54]. However, Liu et al.’s [55] study showed that shading treatment reduced the leaf flavonoid content of *Cyclocarya paliurus*. Hence, different plants have different accumulation patterns of secondary metabolites, including flavonoids, under different light intensities or light qualities. The contents of most secondary metabolites in the anthocyanin biosynthesis pathway (ko00942) and flavone and flavonol biosynthesis pathway (ko00944) decreased with the increase in the shading degree in blueberry, similar to the results from Wang et al.’s [53] study. This study provides a basis for creating shading gradients and examining the metabolite synthesis and accumulation of blueberry in the summer.

## 4. Materials and Methods

### 4.1. Plant Materials

Two-year-old potted ‘Biloxi’ blueberry seedlings with uniform growth and no diseases or pests were selected as the experimental materials. The matrix ratio was 3:3:3:1 peat, coconut bran, pine bark and perlite, and the pH was approximately 5.5. There were three replicates per treatment and 3–4 seedlings per replicate. The seedlings grew in the test base of Nanjing Lishui White Horse Industrial Park, Institute of Botany, Jiangsu Province, Chinese Academy of Sciences (119°03′ E, 31°35′ N). Shade treatments were carried out from the end of July to the end of September using a one-layer black shading net (Taizhou Jinnong Screen Factory, Zhejiang, China) (T1, 50% shading), two-layer black shading net (T2, 80% shading) and no shading (CK, total light). The light intensity changes constantly on any given day in the summer. Under total light, the minimum light intensity was 35,400 lx, and the maximum high light intensity was 88,400 lx. In most cases, the CK light intensity was about 66,100 lx.

### 4.2. Determination of Blueberry Leaf Colour

For blueberry leaf colour measurement, photos were taken under uniform conditions, and the colour parameters were recorded using Adobe Photoshop CS6 software, in which L*, a* and b* values were directly measured by a colour detector. In brief, the middle, upper, lower, left, and right parts of each sample leaf were measured, and four biological replicates were performed [56].

### 4.3. Sample Preparation

Sample preparation was carried out according to previously published research in our laboratory [56]. A total of nine samples were analysed. In brief, 80 mg per blueberry leaf was placed into 20 μL of internal standard (l-2-chlorophenylalanine, 0.3 mg/mL; methanol configuration) and 1 mL of methanol water (*v/v* = 7:3). Then, we added two small steel balls, precooled samples at −20 °C for 2 min, and ground them in a grinder at 60 Hz for 2 min. Ultrasonic extraction was performed in an ice water bath for 30 min followed by incubation overnight at −20 °C. Then, the samples were centrifuged for 10 min (13,000 rpm, 4 °C), and 150 μL of the mixture was aspirated with a syringe. Finally, the mixture was filtered through a 0.22 μm organic phase pinhole filter, transferred to an LC injection vial and stored at −80 °C until LC–MS analysis. All extraction reagents were precooled at −20 °C before use.

### 4.4. Sample Processing

A Dionex Ultimate 3000 RS UHPLC system fitted with a Q-Exactive quadrupole-Orbitrap mass spectrometer equipped with a heated electrospray ionization (ESI) source (Thermo Fisher Scientific, Waltham, MA, USA) was used to analyse the metabolic profiles in both ESI-positive and ESI-negative ion modes. An ACQUITY UPLC HSS T3 column (1.8 μm, 2.1 × 100 mm) was employed. The binary gradient separation system consisted of (a) water (containing 0.1% formic acid, *v/v*) and (b) acetonitrile (containing 0.1% formic acid, *v/v*). The following gradients were used for separation: 0 min, 5% B; 2 min, 5% B; 4 min, 30% B; 8 min, 50% B; 18 min, 80% B; 14 min, 100% B; 15 min, 100% B; 15.1 min, 5% B; and 16 min, 5% B. The flow rate was 0.35 mL/min, and the column temperature was 45 °C. For other sample processing-specific details and parameters, refer to He et al. [57] and Wang et al. [58].

### 4.5. Data Preprocessing

Data preprocessing was conducted according to Wang et al. [58]. In brief, the raw LC–MS data were identified and analysed by Progenesis QI v2.3 software (Nonlinear Dynamics, Newcastle, UK). The precursor tolerance was set at 5 ppm, fragment tolerance was set at 10 ppm, product ion threshold was set at 5% and retention time (RT) tolerance was set at 0.02 min. For statistical analysis of the specific data, please refer to Liu et al. [59]. An Excel file was used for three-dimensional data sets, including m/z, peak RT and peak intensities, and RT–m/z pairs were used as the identifier for each ion.

### 4.6. Statistical Analysis

The PCA and (orthogonal)PLS-DA were carried out to visualize the metabolic alterations among experimental groups after mean centring and Pareto variance scaling, respectively. Variable importance in the projection (VIP) ranks the overall contribution of each variable to the OPLS-DA model, and those variables with VIP > 1 are considered relevant for group discrimination. The DEMs were selected on the basis of the combination of a statistically significant threshold of VIP values obtained from the OPLS-DA model and *p* values from a two-tailed Student’s t test on the normalized peak areas, where metabolites with VIP values larger than 1 and *p* values less than 0.05 were considered DEMs. Moreover, based on the KEGG database, metabolic pathway enrichment analysis of DEMs was performed. A hypergeometric test was used to find the pathway entries significantly enriched in the significantly DEMs compared with the overall background [60].

## 5. Conclusions

In conclusion, moderate shading is conducive to the growth and metabolism of blueberry, but transitional shading will strongly inhibit the accumulation of metabolites in blueberry leaves. Among the top 10 pathways with significant KEGG enrichment across different comparisons, it was found that enrichment of the flavone and flavonol biosynthesis pathway was the most significant. This study showed that blueberry growth and metabolite synthesis and accumulation were optimal under a shading net that allowed, 50% of the summer light. The results provide a reference for the cultivation conditions to obtain high-quality and high-yield blueberry in the summer.

## Data Availability

The data and materials supporting the conclusions of this study are included within the article.

## Supplementary Materials

The following are available online at https://www.mdpi.com/article/10.3390/metabo12010079/s1, Figure S1: The base peak chromatogram diagrams of 9 samples in positive ion mode, Figure S2: The base peak chromatogram diagrams of 9 samples in negative mode, Table S1: The significantly differentially expressed metabolites of blueberry leaves.
