# Supplementary figures and images for: Metabolite Profiling and Classification of Highbush Blueberry Leaves under Different Shade Treatments
Source: Metabolites. 2022 Jan 14;12(1):79. doi: 10.3390/metabo12010079 (PMC8778333; doi:10.3390/metabo12010079)

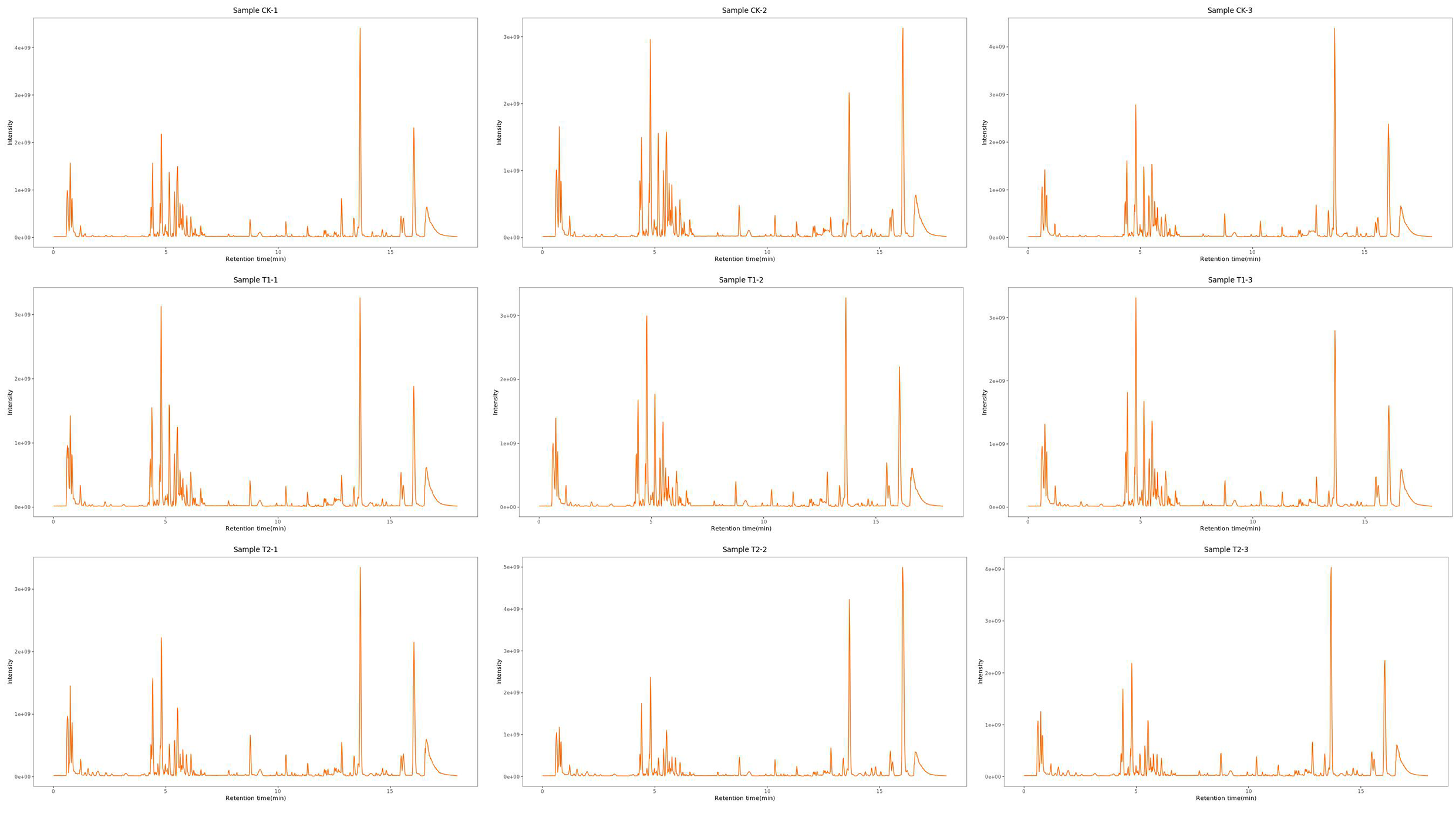

Supplement: Supplementary file 1 [file metabolites-12-00079-s001.zip › SM/FigureS1.tif]

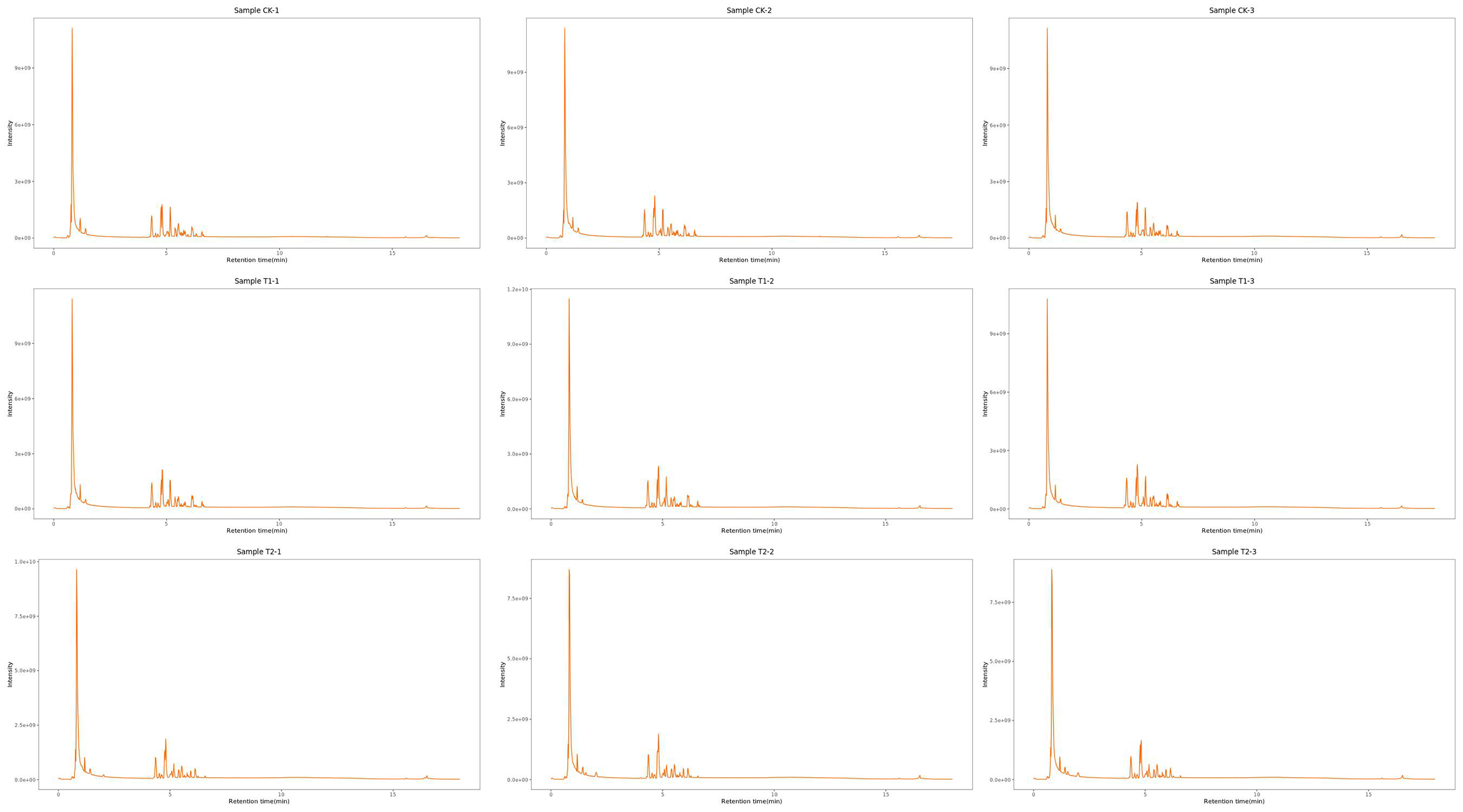

Supplement: Supplementary file 1 [file metabolites-12-00079-s001.zip › SM/FigureS2.tif]
